# Enhanced ANN-based ensemble method for bridge damage characterization using limited dataset

**DOI:** 10.1038/s41598-024-73738-5

**Published:** 2024-10-17

**Authors:** Ivan Izonin, Illia Nesterenko, Athanasia K. Kazantzi, Roman Tkachenko, Roman Muzyka, Stergios Aristoteles Mitoulis

**Affiliations:** 1 Department of Civil Engineering, School of Engineering, University of Birmingham, Birmingham, B15 2TT UK; 2 Department of Artificial Intelligence, Lviv Polytechnic National University, Kniazia Romana str., 5, 79905 Lviv, Ukraine; 3 Department of Publishing Information Technologies, Lviv Polytechnic National University, S. Bandera str., 12, Lviv, 79013 Ukraine

**Keywords:** Cascade ensemble, Bridge, Small data approach, Damage identification, Nondestructive methods, Input-doubling method, Limited data, GRNN, ANN, Data augmentation, Civil engineering, Computer science

## Abstract

Bridges are vital assets of transport infrastructure, systems, and communities. Damage characterization is critical in ensuring safety and planning adaptation measures. Nondestructive methods offer an efficient means towards assessing the condition of bridges, without causing harm or disruption to transport services, and these can deploy measurable evidence of bridge deterioration, e.g., deflections due to tendon loss. This paper presents an enhanced input-doubling technique and the Artificial Neural Network (ANN)-based cascade ensemble method for bridge damage state identification and is exclusively relying on small datasets, that are common in structural assessments. A new data augmentation scheme rooted in the principles of linearizing response surfaces is introduced, which significantly boosts the efficiency of intelligent data analysis when faced with limited volumes of data. Furthermore, improvements to a two-step ANN-based ensemble method, designed for solving the stated task, are presented. By adding the improved input-doubling methods as simple predictors in the first part of the cascade ensemble and optimizing it, we significantly boost accuracy (7%, 0.5%, and 8% based on R2 in predicting tendon losses for three critical zones that were defined across the deck of a real deteriorated prestressed balanced cantilever bridge). This improvement is strong evidence of the accuracy of the proposed method for the task at hand that is proven to be more accurate than other methods available in the international literature.

## Introduction

Bridges are critical components of transport systems, facilitating the mobility of people and goods across geographical barriers^1^. However, the structural integrity of bridges can be compromised over time due to various stressors^2^, leading to damage that not only affects their functionality, but also poses significant risks to public safety and economic productivity. A very recent example of this, is the failure of the Scott Key Bridge in Baltimore, Maryland^3^, that disrupted the operations of the port causing $191 million a day in lost economic activity. Bridge assessment is not always detailed. However, macroscopic evidence, such as excessive deflections, can be utilized for rapid assessments, based on nondestructive methods^4^.

Nondestructive bridge damage state identification techniques play a critical role in evaluating the safety and structural integrity of bridges^5^. These methods offer a means of assessing the structural health of bridges without causing further harm or disruption to their functionality^6^. The field is becoming even more important in view of the ageing infrastructure; a condition that results to increased concerns about their structural integrity and safety. Additionally, in the context of emerging challenges, such as climate change and increased traffic loads, the ability to accurately assess the structural health of bridges through nondestructive means becomes increasingly indispensable^7^.

Nondestructive techniques^8^provide engineers and managing authorities with valuable insights into the condition of bridges, enabling timely maintenance, repair, and retrofitting actions to mitigate potential risks. Such methods have become more sophisticated and effective over the years in accurately identifying various types and degrees of damage in bridges with the advancements in sensor technology, data analytics, and artificial intelligence^9^. However, in cases where there is insufficient data for analysis, the effectiveness of such methods is reduced or even the application of machine learning (ML) techniques for solving the stated problem becomes impossible^10,11^. Such a problem arises quite often because simulation environments for generating datasets operate slowly, while practice engineers are disconnected from the capabilities of ML. Therefore, collecting a sufficient number of observations that would formulate a representative dataset, covering all possible scenarios for a certain type of deterioration mechanism, is almost impossible considering the tight budgets and resources in analysis and assessment.

In Kazantzi et al^12^., the application of ML methods for damage state identification in deteriorating bridges based on a limited dataset was investigated. The particularity of the problem in^12^ is the need to predict damage (in the form of tendon losses) in three different zones, that were defined across the deck of a balanced cantilever bridge, that are interdependent. The authors collected a dataset of 81 observations using a simulation environment and utilized the k-nn method to address the problem. Considering the specificity of the task and the limited data available for training, the results obtained are relatively satisfactory yet there is certainly room for further improvement in the predictions made by the proposed methodology. Therefore, there is a need to improve the predictive accuracy of the developed method.

In Izonin et al^13^., the aforementioned stated problem is treated as a regression task. The authors developed a two-level cascade method using General Regression Neural Networks (GRNN) as weak predictors to address two tasks: (a) effective intelligent analysis in the case of limited data and (b) consideration of interdependencies among the three output attributes to be predicted. Despite the increased accuracy of the proposed cascade compared to the method that was initially proposed in Kazantzi et al^12^., GRNN as a weak predictor may not provide sufficient efficiency for solving the stated problem. This could lead to a decrease in the accuracy of the entire cascade and the need for improvement. Increasing the number of observations in the training sample may mitigate the aforementioned drawback^10,14^. However, obtaining the additional observations requires significant time, human and computational resources that are not always available in practical engineering applications.

To address this challenge, Izonin et al^15^.. developed an input-doubling method that quadratically increases the volume of training data, which could potentially improve the accuracy of GRNN’s operation. However, this method demonstrates only a negligible increase in accuracy compared to the increased operation duration due to the augmentation procedure. Moreover, this method can predict only one output, whereas in the investigated task three interconnected outputs need to be predicted. Nevertheless, this method especially its improvement can increase the prediction accuracy for one output and therefore can be used as weak regressors for the proposed method^13^.

This paper improves the accuracy of the bridge damage state identification task founded upon a limited dataset, by enhancing an existing two-step ANN-based ensemble method. This was accomplished through the utilization of enhanced input-doubling methods based on GRNN as weak predictors in the cascade ensemble.

The main contributions of this paper can be summarized as follows:

1. The input-doubling method for small data analysis was enhanced by employing a new data augmentation scheme based on the principles of linearizing response surfaces, resulting in increased efficiency of intelligent data analysis with limited volumes.
2. A two-step ANN-based ensemble method for solving the bridge damage state identification task was enhanced by utilizing improved input-doubling methods as weak predictors in the first step of the cascade ensemble, leading to a significant improvement in the accuracy of solving the stated task.
3. The enhanced ANN-based ensemble method was optimized and achieved the highest prediction accuracy for a damage state identification task of a real-world case-study bridge using a small dataset for training (81 vectors derived from 27 cause-agnostic scenarios of tendon loss, with four input attributes) compared to a series of existing methods.

The paper is organized as follows. In “Materials and methods” section describes the dataset for modeling, the topology, and the working principles of the GRNN. Additionally, it includes a structural-functional diagram as well as training and application algorithms of the enhanced input-doubling method for the analysis of a limited dataset, that is used as weak predictors for the improved cascade scheme for bridge damage state identification. It also presents a structural-functional diagram and describes the training and application algorithms of the enhanced ANN-based ensemble method that is the core of this paper. The third section of the paper is dedicated to modeling, selecting optimal parameters, and the results of the improved methods. It also compares the performance of the latest methods with several existing ones. In “Conclusions” section of the paper describes the implications of the proposed work. The conclusions of the work are presented in the fifth section of the paper.

## Materials and methods

This section outlines the peculiarities of the stated problem, describes the datasets for modeling (obtained from the simulations of several damaged scenarios of an actual case-study bridge), and constructs the corresponding GRNN topology, which forms the basis of the two enhanced methods. Two improved methods are described, their operation is visualized, and the main steps of the algorithms for their training and application are outlined.

### Dataset description

Bridge deterioration often leads to excessive deflections, indicating potential structural damage. Despite the existence of various nondestructive monitoring methods for detecting geometric changes in bridges, such as deck deflections and foundation settlement, there is a lack of methods for linking these measurements with specific levels of damage. This knowledge gap is crucial for devising effective restoration strategies, which directly impact costs and intervention scopes.

Kazantzi et al^12^. developed a dataset of numerically simulated and validated structural responses to address this issue. In particular, several variations of a bridge finite element model were developed to simulate different damage scenarios, related to tendon losses, that were assumed to be the dominant failure mode for the utilized case-study application, i.e., a prestressed balanced cantilever bridge. To facilitate an easier definition of the tendon loss damage scenarios the cantilever part of the bridge was subdivided into three Zones. Vertical displacements of the bridge deck, expressed as drifts along its length in a non-dimensional manner, were consequently computed. As a result, the training dataset comprises 81 observations, derived from 27 cause-agnostic scenarios of tendon losses, with four input attributes (Vertical Drift Ratios computed at three characteristic locations along the bridge deck, that approximately correspond to the end of defined deck Zones 1, 2, and 3 and the Concrete Young’s Modulus) and three interconnected output attributes (Tendon Losses in Zones 1, 2, and 3).

A test dataset, consisting of five observations for each Zone, is utilized for validating the proposed ML solution. Both datasets from this research study^12^ are employed for intellectual analysis and validation in this paper.

### General regression neural network

The General Regression Neural Network (GRNN) was developed quite a while ago and it is frequently used in solving various prediction and forecasting tasks. GRNN serves both as a data preprocessing tool and as a primary instrument for solving different tasks in the civil engineering field^16^. The popularity of this Artificial Neural Network (ANN) is attributed to its simplicity and high efficiency in analyzing small datasets in various application domains. GRNN, as an Radial Basic Functions (RBF)-like ANN, possesses several advantages, including superior generalization, a single-pass learning mode, the need of only one parameter that requires configuration, etc^13,17,18^.

**Fig. 1:**
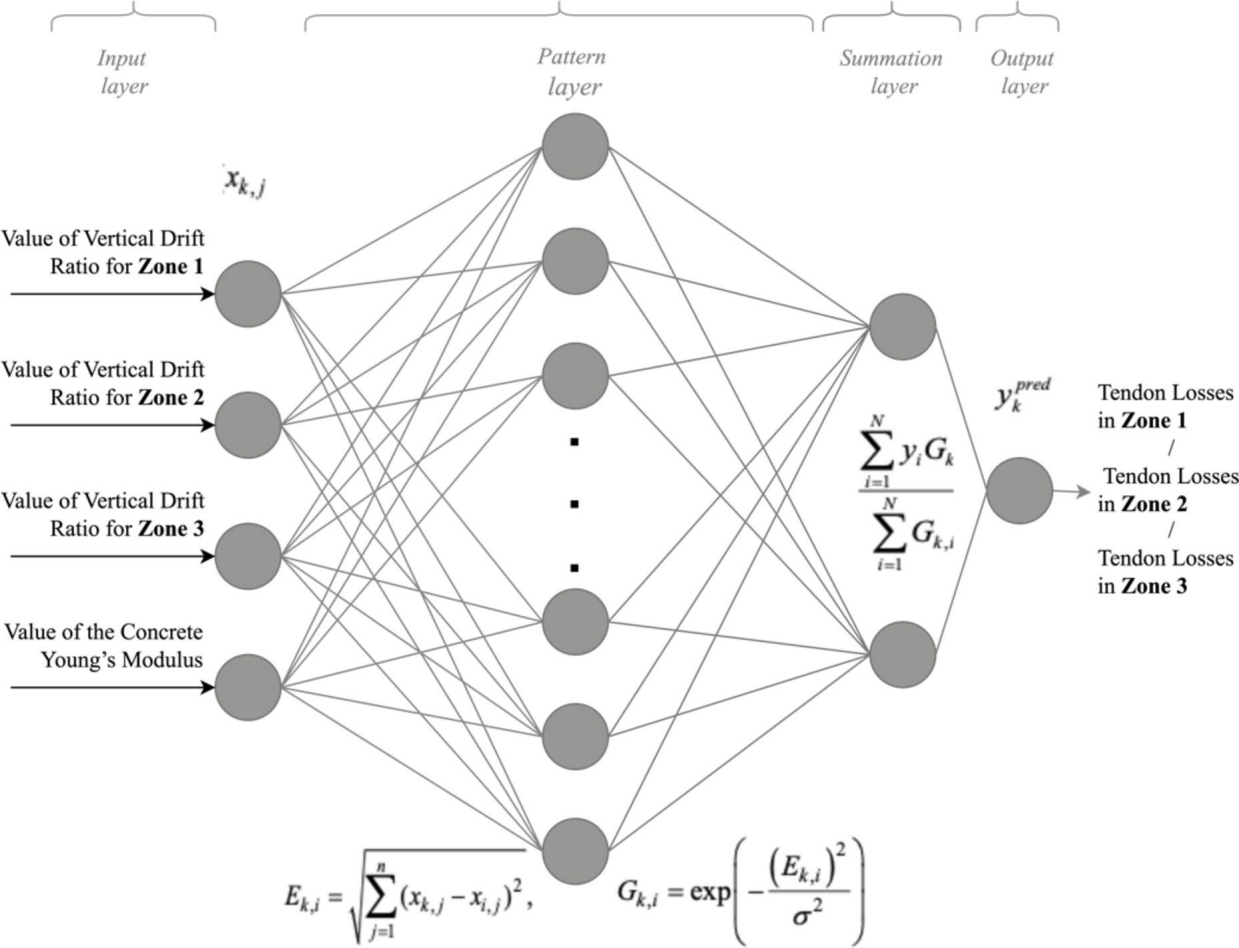
Topology of the GRNN for solving the bridge damage state identification task.

The topology of GRNN for solving the problem addressed by this paper is depicted in Fig. 1.

It comprises four inputs in the input layer, corresponding to four independent attributes. Additionally, there are pattern and summation layers, with the calculations provided by the formulas in Fig. 1, and the output layer. In our case, the output signal of GRNN is the value of one of the three dependent attributes that need to be found.

## An enhanced input-doubling method for the analysis of a limited dataset

Despite the several advantages of the GRNN, this type of neural network does not always provide high prediction accuracy^15^. To address this drawback, a method based on input doubling has been proposed in Izonin et al^15^., which is grounded on the principles of ML based on the axial symmetry of the response surface. This method quadratically expands the given small dataset, which should increase the prediction accuracy. However, a significant drawback of this method is the substantial increase in the operation time due to the expansion of the independent attributes of the problem (doubled inputs) and the use of the authors’ data augmentation procedure (quadratic increase in the dataset). This is particularly noticeable for cases where the initial dataset contains more than 100 vectors.

The authors of the aforementioned method^15^ managed to achieve an improvement in the prediction accuracy based on the analysis of a small dataset. However, this improvement is relatively small considering the significant increase in the method’s operation time. Therefore, in this paper, we propose an enhancement of this method, which is based on the principles of linearizing the response surface.

**Fig. 2:**
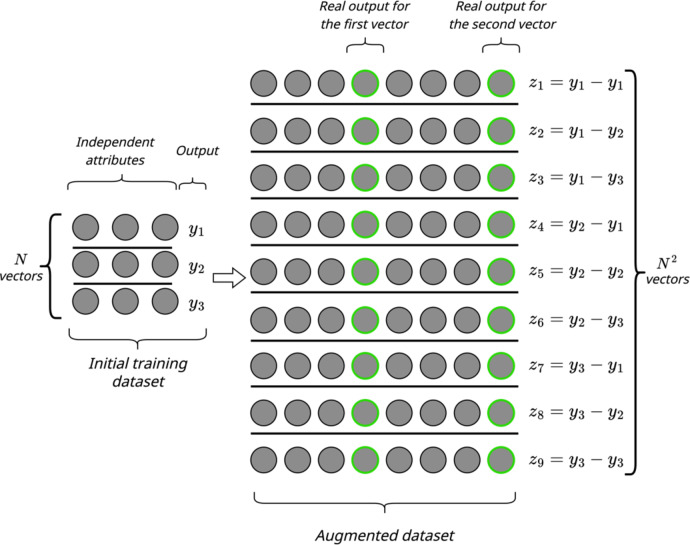
Data augmentation procedure for training GRNN of the enhanced input-doubling method.

**Fig. 3:**
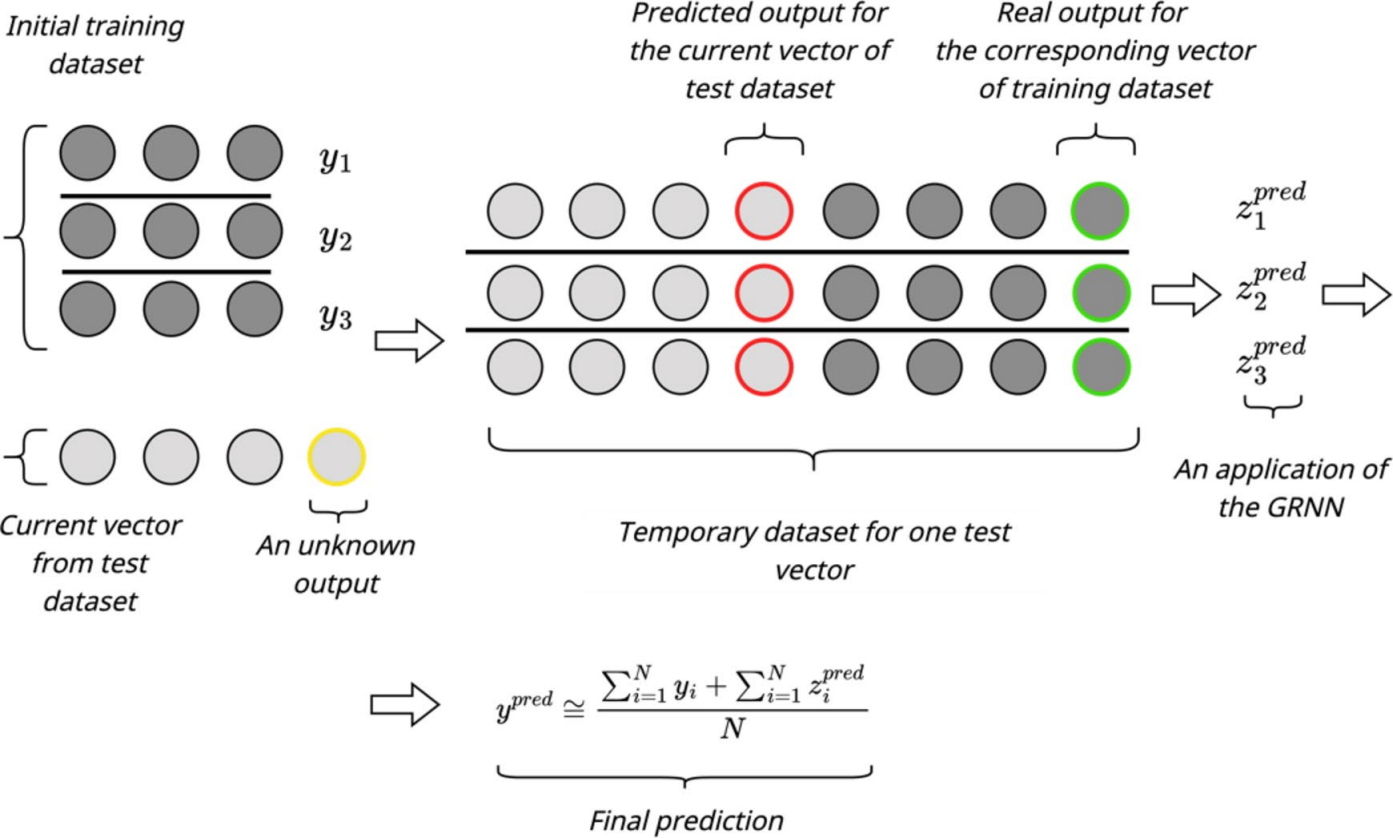
Application procedure of the enhanced input-doubling method.

Similarly to the basic method in^15^, the enhanced input-doubling method involves the following main procedures: data preparation (augmentation), applying GRNN, and using the authors’ procedure for forming the predicted value. The flowcharts of both procedures are illustrated in Figs. 2 and 3, respectively.

In more detail, the data preparation procedure is as follows. The basic method in^15^involves concatenating two vectors from the given small dataset. This procedure is performed for all available vectors in the training set. As a result, a quadratically expanded new dataset is obtained with a doubled number of features in each new vector. According to the enhancement proposed in this paper, two additional elements are suggested to be added to each doubled vector from the new dataset - the existing output values of the corresponding vectors being concatenated. Thus, the dimensionality of the problem is increased by two attributes, while still maintaining the quadratic expansion of the given dataset. The dependent attribute for each such pair is formed as the difference between the outputs of the two current vectors forming the new extended data vector^15^. This new dataset is used as the training set (or in our case, as the support set) for the operation of the GRNN method.

The procedure for applying the enhanced method requires the availability of: *(i)* the initial dataset, *(ii)* the quadratically augmented support set, and *(iii)* the current vector with an unknown output value. In the first step of the enhanced method, the unknown output value of the current vector of the test set is predicted using the GRNN_1 method. It should be noted that in this case, GRNN_1 uses the initial training dataset as the support set. Next, the current vector is concatenated with each vector of the initial training dataset. In addition to concatenating independent features, according to the enhancement proposed in this paper, two new attributes are added to each augmented vector. The first one is the actual output value of the corresponding vector from the initial training set. All of them are known. The second one is the predicted value of the desired output for the current vector of the test set using the GRNN_1, as it is unknown.

Through performing the above actions, we obtain a temporary dataset for the current vector of the test set. Using the new GRNN_2 and the new augmented dataset from Fig. 2, we predict the output signals for each vector of the temporary dataset from Fig. 3. Then, after obtaining the output signals and having the known output values for the initial support set, the final prediction value for one vector of the test set according to the formula presented in Fig. 3^15^is being searched. These procedures are repeated for each new vector with an unknown output signal (all other vectors of the test set)^15^.

Utilizing such an enhanced method, that is built upon the principles of linearization of the response surface, is anticipated to significantly increase the prediction accuracy when analyzing small datasets, both compared to the basic input-doubling method as well as compared to the existing GRNN.

## An enhanced ANN-based ensemble method for bridge damage state identification using a limited dataset

At the core of the enhanced ANN-based cascade method for solving the bridge damage state identification task lies the existing method from^13^. It was specifically developed to address the task of predicting three interdependent output attributes. For this purpose, the ensemble method from^13^involves two steps, each utilizing three GRNNs to predict the tendon losses at the three different Zones (Zone 1, Zone 2, and Zone 3) of the bridge deck. Both of the aforementioned steps are executed sequentially, thus forming a cascading ensemble with two levels. Six weak predictors, namely six GRNNs, are used according to^13^to provide satisfactory accuracy in solving the stated task, considering the small amount of data available for analysis. However, as noted by the authors in^13^, using a larger amount of data may ensure higher accuracy and a more reliable prediction outcome.

**Fig. 4:**
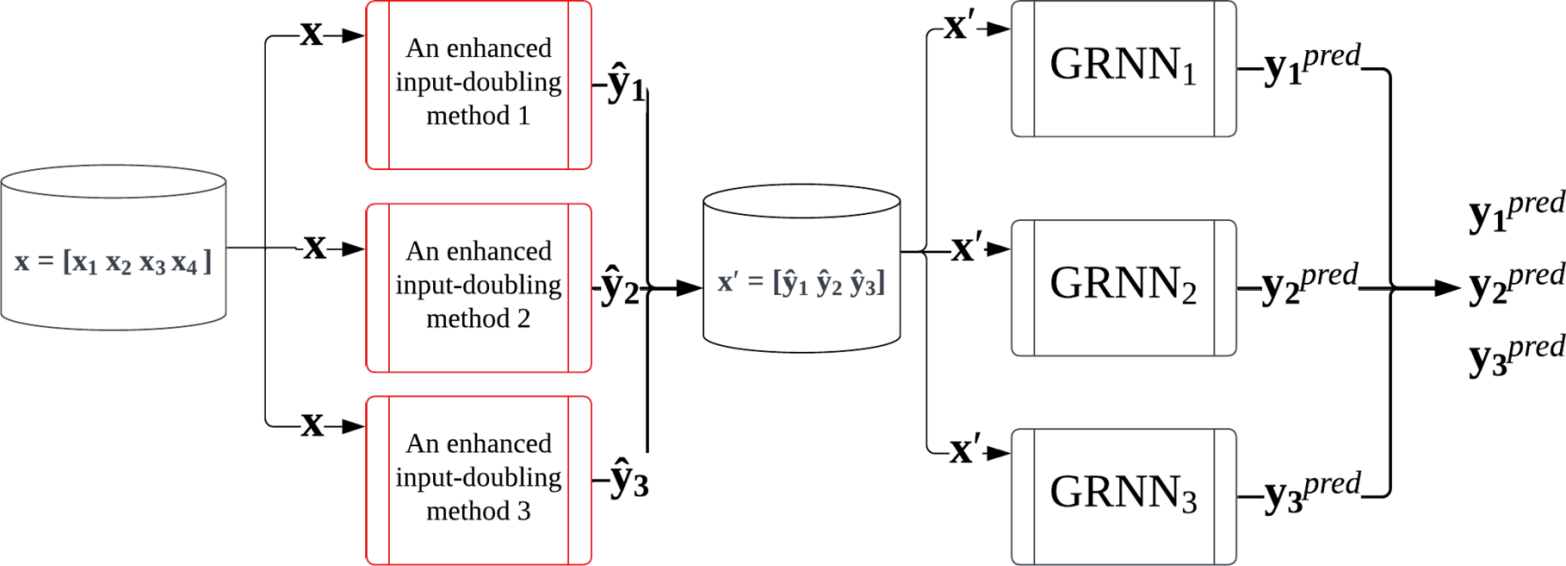
Application procedure of the enhanced ANN-based ensemble method.

Therefore, in this paper, it is proposed to use the enhanced input-doubling methods as weak predictors in the first step of the method. Such an approach should ensure an increase in prediction accuracy by utilizing a larger amount of data based on the author’s data augmentation procedure. The flowchart of the enhanced ANN-based ensemble method for solving the bridge damage state identification task using a limited dataset is presented in Fig. 4.

The notations used in the enhanced ANN-based ensemble method from Fig. 4 are presented in Table 1.

**Table 1:** The main notations used in Fig. 4.

№	Notation	Explanation
1	$x=\left[ {{x_1},{x_2},{x_3},{x_4}} \right]$	The dataset with the primary 4 inputs attributes
2	$\widehat {{{y_i}}},~i=1,2,3$	The predicted output of the enhanced input-doubling method for the *i*-Zone after first level of the enhanced ensemble
3	The enhanced input-doubling method	A weak predictor at the first level of the enhanced cascade ensemble
4	GRNN	General Regression Neural Network – a weak predictor at the second level of the enhanced cascade ensemble
5	$\acute{x}=\left[ {{{\hat {y}}_1},{{\hat {y}}_2},{{\hat {y}}_3}} \right]$	New training dataset for the second level of the cascade ensemble that contains predicted output of the enhanced input-doubling method for each 3 Zones
6	$y_{i}^{{pred}},~i=1,2,3$	The final predicted output of the enhanced ANN-based ensemble method for the *i*-Zone

The main steps of applying the enhanced ANN-based ensemble method for bridge damage state identification are the following:

***Step 1:*** Form three datasets differing only in the dependent feature (three different tendon losses in three different Zones). Four independent attributes will be identical across the three datasets.

***Step 2:*** Apply the enhanced input-doubling methods to the three datasets to predict the tendon losses for three different Zones of the first level of the cascade.

***Step 3:*** Create three new datasets by replacing the initial four independent attributes of the task with new ones, each containing only three attributes - the predicted values of the tendon losses for three different Zones obtained in the previous step.

***Step 4:*** Utilize three different GRNNs of the second level of the ensemble on the three new datasets from the previous step to forecast the final values of the tendon losses for three different Zones.

It should be noted that after Step 2, we obtained a new dataset $\acute{x}=\left[ {{{\hat {y}}_1},{{\hat {y}}_2},{{\hat {y}}_3}} \right]$ for training, which contains as input attributes the predicted values $\widehat {{{y_i}}},~i=1,2,3$ of tendon losses for 3 Zones for final processing by neural networks at the next level of the cascade (Step 4). In this way, we take into account that the three output attributes to be predicted are interconnected, thereby increasing the accuracy of their prediction.

## Modeling, results and discussion

Modeling of the enhanced ANN-based ensemble method as well as the enhanced input-doubling method, was conducted using custom software solutions in Python. Experimental investigations were conducted on a computer with the following specifications: CPU Intel(R) Core(TM) i7-8750 H CPU @ 2.20 GHz, RAM 16.0 Gb, GPU NVIDIA GeForce GTX 1050 Ti, 64-bit OS.

Obtained results based on different performance indicators as well as the optimal value of the GRNN’s smooth factor are presented in Table 2.

**Table 2:** Prediction results for each 3 zones using the enhanced ANN-based ensemble method based on different performance indicators.

Zone # / Performance indicators	Zone 1	Zone 2	Zone 3
R^2^	0,943	0,996	0,932
MSE	5,609	0,285	3,224
RMSE	2,368	0,534	1,796
MAE	1,904	0,392	0,870
MAPE	0,044	0,014	0,209
MedError	1,504	0,351	0,001
MaxError	3,662	0,872	4,000
Optimal value of smooth factor	0,129993698	0,030762375	0,030714376

To assess the accuracy of the enhanced method, the paper compares its performance with several existing methods, including:

1. the classical GRNN^19^,
2. the extended-input GRNN^20^,
3. the basic input-doubling method^15^,
4. the basic ANN-based ensemble model^13^.

**Fig. 5:**
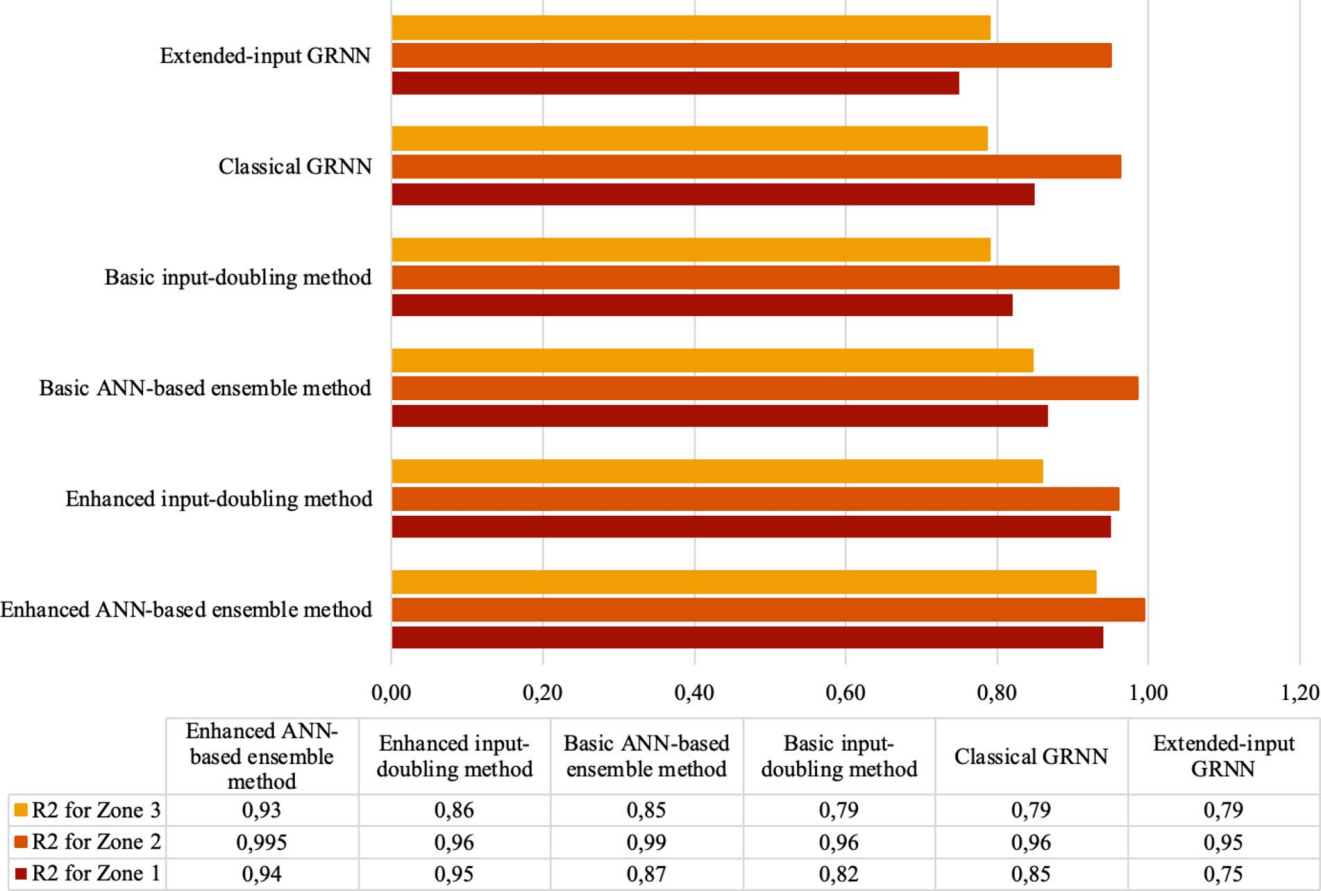
Comparison of different methods (based on the R^2^) for solving the bridge damage state identification task in the three deck Zones.

This selection is motivated by the fact that all these methods are based on the utilization of GRNN. To optimize the performance of each of the investigated methods, the authors employed the differential evolution method^21^. As investigated in^21^, this method facilitates rapid search for the optimal value of a single GRNN’s parameter that influences its accuracy. In comparison with the dual annealing method, differential evolution provides the same results but much faster. This was the main criterion for choosing this method to select the optimal parameter for the operation of the GRNN. All obtained results based on the coefficient of determination (R^2^)^22^ are summarized in Fig. 5.

As could be seen in Fig. 5, the lowest accuracy results for all three zones are demonstrated by the classical GRNN^19^, extended-input GRNN^20^, and basic input-doubling method^15^. This can be explained by their failure to consider necessary information in the form of two additional attributes for prediction, which are contained in the tendon losses of both Zones during the prediction of the third. Significantly better results were shown by the basic ANN-based ensemble model^13^ because it mitigates the aforementioned limitation of the three existing methods by utilizing a two-level cascade structure. It accounts for the interdependencies of the three output attributes and provides all available independent attributes to weak regressors of the second level to enhance prediction accuracy.

The enhanced input-doubling method presented in this paper demonstrated higher accuracy compared to the basic ANN-based ensemble model^13^ by 7%, 0%, and 13% for all three Zones, respectively. This can be attributed to a substantial increase in the training dataset size due to the author’s augmentation procedure and the utilization of principles of response surface linearization. However, this method is not reliable as it fails to consider that the three output attributes are interdependent, thus not leveraging all available information for prediction procedures.

The highest accuracy across all three zones was achieved by the enhanced ANN-based ensemble approach presented in this paper. Specifically, its usage in an accuracy increase based on R^2 ^by 7%, 0.5%, and 8% for Zone 1, Zone 2, and Zone 3, respectively, compared to the best method for solving the stated task (basic ANN-based ensemble model^13^). This reveals several advantages of utilizing the enhanced ANN-based ensemble model presented in this paper for solving the damage state identification task for other bridges.

When discussing the computational resources^23^ required for the enhanced methods presented in the paper, it is evident that there has been a significant increase. Table 3 summarizes the execution times for all the studied methods used to solve the stated task. It is important to note that all the methods investigated are based on GRNN, which does not require a training procedure.

**Table 3:** Application time for all investigated methods (in seconds).

Method	Zone 1	Zone 2	Zone 3
Enhanced ANN-based ensemble method	356,19	590,35	1107,92
Enhanced input-doubling method	356,10	590,07	1107,76
Basic ANN-based ensemble method^13^	1,25	1,53	2,13
Basic input-doubling method^15^	294,16	175,25	486,09
Classical GRNN^19^	0,10	0,12	0,07
Extended-input GRNN^20^	0,19	0,39	0,81

As shown in Table 3, the Enhanced Input-Doubling Method, improved in this study, exhibits a significant increase in the duration of the inference procedure compared to the baseline ANN-based ensemble method^13^. This increase can be attributed to two main factors. Firstly, the extended dataset requires processing a greater number of features. Secondly, the Enhanced Input-Doubling Method involves using GRNN twice, necessitating additional time to select optimal values for both smooth factors to ensure the best performance of both neural networks.

When examining the Enhanced ANN-Based Ensemble Method, which employs three Enhanced Input-Doubling Methods at the first level of the cascade, its execution time also significantly increases compared to the Basic ANN-Based Ensemble Method^13^. This increase is due to the use of three Enhanced Input-Doubling Methods instead of the Classical GRNN^19^. However, as the accuracy results indicate, this combination provides substantially lower errors in solving the task outlined in the paper. Additionally, this study focuses on the analysis of small datasets for solving the specified problem, which generally do not require extensive processing time. Given that the paper discusses a non-destructive method for diagnosing bridge structures, the approximate six-minute processing time is justified compared to the resource-intensive, destructive methods typically used for such diagnostics.

The proposed study on bridge damage state identification^24^using enhanced ANN-based ensemble method has significant implications across several dimensions. First of all, the proposed enhancement of the input-doubling technique and the ANN-based cascade ensemble method represents a methodological advancement. These improvements enable more accurate and efficient bridge damage state identification, especially when dealing with limited datasets, which is common in real-world applications^25^. Such method’s class (nondestructive methods) are pivotal as they allow for assessing bridge conditions without causing further harm or disruption. Enhancing these methods improves efficiency and reduces maintenance costs associated with traditional inspection methods^26^.

Secondly, the validation of the method in identifying specific bridge damages in Greece highlights its real-world applicability and effectiveness compared to existing methods. The demonstrated improvements in accuracy, such as the 7%, 0.5%, and 8% enhancements in predicting tendon losses for different zones, underscore the practical benefits of the proposed approach. This practical demonstration suggests potential broader applicability in various geographical and operational contexts.

The proposed ANN-based ensemble method demonstrates high efficiency in analyzing extremely short datasets for training (up to 100 vectors). When considering datasets of medium size (from 100 to 1000 vectors), the method also operates effectively. In this case, there is no need to apply data augmentation procedures, making classical GRNNs suitable as weak predictors at both levels of the ensemble. They ensure high prediction accuracy with minimal computational resources required for their operation. Scaling the method to large datasets maintains the composition of the method under these circumstances. The exception would be the choice of appropriate weak predictors at both levels of the cascade ensemble. In such cases, high-speed and high-precision methods like ensemble machine learning methods^11^, should be selected to ensure efficient data processing at both levels of the cascade ensemble. However, it should be noted that collecting a large amount of data to form a training sample for solving the stated task is time-consuming and resource-intensive. Therefore, the improved method presented in this paper, that is capable of effectively handling the analysis of extremely small training datasets, holds significant practical value.

## Conclusions

This paper has underscored the pivotal role of rapid bridge damage characterization in safeguarding the safety of such vital transportation infrastructure assets globally. By leveraging nondestructive methods, we have presented significant advancements in enhancing the accuracy and efficiency of bridge damage assessment, particularly when dealing with limited datasets.

The introduction of a novel data augmentation scheme, rooted in principles of linearizing response surfaces, has proved instrumental in bolstering the efficacy of intelligent data analysis in the face of constrained data volumes. Moreover, improvements to the two-step ANN-based ensemble method, facilitated by the integration of enhanced input-doubling techniques and subsequent optimization efforts, have yielded notable enhancements in accuracy.

By means of an actual bridge asset, the proposed solution has demonstrated superior performance compared to the existing ANN-based cascade method, as evidenced by achieved improvements of 7% and 8% (based on R^2^) in predicting tendon losses in Zone 1 and Zone 3, respectively. These results underscore the effectiveness of the proposed approach in addressing the complexities of bridge damage identification tasks.

Looking ahead, future research endeavors will focus on refining and iterating upon the proposed methodologies to further enhance their prediction accuracy. Additionally, investigations into the influence of different ANN architectures as weak predictors within the cascade ensemble scheme will be pursued, offering valuable insights into optimizing model performance.

## Data Availability

The data that support the findings of this study are available from the corresponding author, Ivan Izonin, upon reasonable request.
